# The Vigilance Gradient: Eleven Years of Adverse Event Trends in Pediatric Critical Care

**DOI:** 10.1097/CCE.0000000000001407

**Published:** 2026-04-24

**Authors:** Devika Singh, Michael R. Miller, Maitray A. Patel, Cory Anderson, Douglas D. Fraser

**Affiliations:** 1 Department of Pediatrics, Western University, London, ON, Canada.; 2 Children’s Health Research Institute, London, ON, Canada.; 3 Epidemiology and Biostatistics, Western University, London, ON, Canada.; 4 GSK Chair in Clinical Pharmacology, Western University, London, ON, Canada.

**Keywords:** adverse events, patient safety, pediatric critical care, quality improvement, risk stratification

## Abstract

**OBJECTIVES:**

To examine 11-year trends in adverse events (AEs) in a pediatric critical care unit (PCCU), assess the impact of the COVID-19 pandemic on patient safety, and evaluate associations between patient acuity and AE severity.

**Design:**

Retrospective cohort study using interrupted time series analyses and mixed-effects multinomial regression.

**Setting:**

A single-center PCCU monitored via the Adverse Event Management System from January 2013 to December 2023.

**Patients:**

A total of 7290 critically ill and injured pediatric patients admitted to the PCCU over the study period.

**Interventions:**

None (observational study). Exposure variables included the COVID-19 pandemic period, invasive mechanical ventilation and noninvasive ventilation.

**Measurements and Main Results:**

Demographics, length of stay (LoS), disposition, and AE severity were assessed. The baseline AE rate was 11.94 events per 100 cases. At pandemic onset, AE rates rose by 5.20 events per 100 cases (*p* = 0.004), then declined 0.81 events per 100 cases quarterly (*p* = 0.010). Post-pandemic, rates increased 1.94 events per 100 cases quarterly (*p* = 0.009). LoS decreased 0.01 days quarterly pre-pandemic (*p* = 0.009), was stable during the pandemic, then increased 0.25 days quarterly post-pandemic (*p* = 0.033). Higher Pediatric Index of Mortality 2 scores were associated with fewer “near miss” events and more “MinModSev” (minimal, moderate, or severe) AEs. Both mechanical ventilation (*p* = 0.039) and noninvasive ventilation (*p* = 0.015) increased the odds of “MinModSev” AEs.

**CONCLUSIONS:**

This PCCU experienced a transient increase in AEs during COVID-19, followed by recovery and a post-pandemic rise in both AE rates and LoS. Higher illness severity and respiratory support were associated with more severe AEs. These findings underscore the importance of data-driven monitoring systems to sustain patient safety during and after healthcare crises.

**KEY POINTS**

**Question**: Does long-term surveillance of adverse events in a pediatric critical care unit demonstrate temporal trends and associations between illness severity and adverse event severity?

**Findings**: In this retrospective cohort study spanning 11 years, higher illness severity and mortality risk were associated with lower adverse event severity, demonstrating a statistically significant “vigilance gradient.” Adverse event patterns also changed during the COVID-19 pandemic.

**Meaning**: These findings suggest that risk-stratified vigilance may mitigate adverse event severity in high-risk pediatric critical care patients and can inform targeted patient safety strategies.

Ensuring the safety of critically ill children remains the cornerstone of pediatric intensive care. Despite remarkable advances in medical technology and treatment protocols, adverse events (AEs) continue to pose significant challenges in these high-acuity environments. The intensive nature of pediatric critical care units (PCCUs) places patients at elevated risk for AEs, ranging from medication errors and procedural complications to infections and ventilation-related issues. These incidents not only compromise patient safety but also prolong hospital stays, increase healthcare costs, and worsen clinical outcomes (1). Understanding long-term patterns of AEs is crucial for developing targeted interventions, yet comprehensive longitudinal analyses in pediatric critical care remain limited.

Existing research has identified key contributors to AEs in PCCUs, including medication administration errors, procedural mishaps, and device-related complications, with certain high-risk patient groups, such as surgical patients and those requiring long-term mechanical ventilation, being particularly vulnerable (2, 3). To address these challenges, institutions have increasingly adopted standardized safety tracking systems like the Adverse Event Management System (AEMS) to facilitate real-time monitoring, standardize AE reporting, and support proactive risk reduction strategies (4–6). However, the long-term effectiveness of these systems, particularly during healthcare crises, requires systematic evaluation.

The COVID-19 pandemic served as an unprecedented stress test for healthcare systems worldwide, fundamentally altering clinical workflows and resource allocation patterns. This global crisis strained healthcare resources and potentially exacerbated AE rates due to increased patient volumes, staffing shortages, and rapid shifts in clinical practice (7, 8). While studies have documented elevated AE rates in adult ICU populations during the pandemic, the specific impact on PCCUs remains poorly characterized (4, 9–11). Despite the introduction of national patient safety frameworks, such as Canada’s Never Events list, institutional-level analyses of long-term AE trends in pediatric critical care settings are notably absent from the literature (5, 12).

This study aimed to fill this critical knowledge gap by analyzing trends in AEs reported over a 9-year period within a PCCU, with particular focus on AE frequency, length of stay (LoS), patient demographics, clinical interventions, disposition patterns, and changes observed during and after the COVID-19 pandemic. By examining fluctuations in AE types and frequency over time, including a natural experiment provided by the pandemic, we sought to provide actionable insights into patient safety in pediatric critical care. Our findings build upon existing knowledge by offering the first comprehensive analysis of long-term AE trends spanning a global healthcare crisis, providing evidence-based foundations for targeted interventions to enhance care quality and reduce patient harm.

## METHODS

### Study Setting and Design

This study was conducted in the PCCU at London Health Sciences Centre, a tertiary-level care center in London, Ontario, Canada. The PCCU at the Children’s Hospital serves as the Regional Pediatric Level 1 Trauma Centre for Southwestern Ontario, providing care to a pediatric population of over 50,000 within a catchment area spanning 190,000 km^2^ (13). As a secondary data analysis involving no direct patient contact, a waiver of consent was granted, and all data were anonymized before analysis.

This study was reviewed and approved by the Western University Health Science Research Ethics Board (Approval No. 123974; approved January 4, 2024; Study Title: “Identification and trends of adverse events in patients admitted to a Pediatric Critical Care Unit (PCCU).” Given the retrospective design and use of existing clinical data, the requirement for informed consent was waived by the Research Ethics Board. All procedures performed in this study were conducted in accordance with the ethical standards of the responsible institutional and/or regional committee on human experimentation and with the Helsinki Declaration of 1975.

### Data Collection and Study Population

We collected retrospective data spanning 11 years (from January, 2013, to December, 2023), encompassing 7290 patients and all AEMS reports filed for PCCU admissions. The AEMS was launched in September 2012 at this institution, allowing our analysis to capture trends from system implementation through the study initiation. Inclusion criteria comprised all PCCU admissions during the study period, with no exclusion criteria applied to ensure comprehensive assessment.

Data were obtained from the AEMS, an internal reporting system designed to track safety incidents within the PCCU. The AEMS is a hospital-wide safety event reporting system used for all patient safety concerns. Reporting is voluntary but strongly encouraged and is embedded within the PCCU safety culture. All staff, including physicians, nurses, respiratory therapists, and allied health professionals, have access to submit reports, and all reported events are reviewed through established quality and safety processes. This ensures that the AEMS captures a broad range of safety concerns, although voluntary reporting may result in underreporting of some events. The AEMS database serves as a structured repository where healthcare providers, including physicians, nurses, respiratory therapists, and allied health professionals, document AEs occurring in patient care. AEMS reports capture comprehensive details including temporal and spatial information, incident nature and category, harm occurrence, individuals involved, contributing factors, interventions implemented, and communication with patients or their substitute decision makers (14). To supplement these data, patient-level clinical information was extracted by the institution’s Decision Support Analysis team, providing demographics, admission dates, LoS, and discharge disposition.

### Adverse Event Classification

AEs were categorized into four severity levels within the AEMS framework: 1) near misses, incidents that did not result in harm; 2) minimal events with no injury, occurrences that did not cause patient harm; 3) moderate events requiring intervention, incidents necessitating additional clinical actions to mitigate potential harm; and 4) severe events resulting in patient harm or critical deterioration.

### Statistical Analysis

Categorical variables were summarized using frequencies and proportions. To assess longitudinal trends in AEs and LoS, interrupted time series (ITS) analyses were conducted using autoregressive integrated moving average models on quarterly data. The timeline was divided into three distinct periods: pre-pandemic (January 2013–March 2020), pandemic (March 2020–December 2023), and post-pandemic (March 2022–November 2023). Total AEs were expressed per 100 PCCU admissions to facilitate interpretation and temporal comparison. The ITS models evaluated changes in both level (immediate onset effects) and slope (trend changes) for AE and LoS patterns across each time period. Autocorrelation functions guided model selection and accounted for cyclical variations. White noise and stationarity were assessed using the Ljung-Box chi-square statistic and Dickey-Fuller test, respectively.

Generalized mixed-effects multinomial regression models examined predictors of AE severity, categorized as near miss, no intervention required, intervention required, and mild-to-severe AEs. In all regression models, AE severity served as the outcome variable, with predictors entered as fixed effects and patient as a random effect using a scaled identity covariance structure with random intercept. Predictors included time period (pre-pandemic, pandemic, post-pandemic), age categories (infancy [0–1 yr], toddler/early childhood [2–5 yr], middle childhood [6–11 yr], adolescence [12–17 yr]), disposition (died, discharged, transferred to inpatient unit, transferred to another institution, left against medical advice), severity scores (Pediatric Index of Mortality 2 [PIM2], Pediatric Logistic Organ Dysfunction [PELOD], Nine Equivalents of Nursing Manpower Use Score [NEMS]), and clinical interventions (central venous line, arterial line, IV medications, multiple IV medications, intracranial pressure [ICP] monitoring, mechanical ventilation, noninvasive ventilation, bilevel positive airway pressure [BiPAP], continuous positive airway pressure [CPAP], high-flow nasal cannula; **Table 1**).

**TABLE 1.** Association Between Respiratory Support Modality and Adverse Events, and Distribution of Pediatric Index of Mortality 2 Scores by Adverse Event Severity

Respiratory Support Type		OR (95% CI)		*p*
Mechanical ventilation		2.85 (2.68–3.03)		< 0.001
Noninvasive ventilation		1.11 (1.06–1.15)		< 0.001
Bilevel positive airway pressure		0.998 (0.909–1.095)		0.960
Continuous positive airway pressure		0.998 (0.909–1.096)		0.972
High-flow nasal cannula		1.007 (0.923–1.099)		0.872
Adverse Event Severity Category	*n*	PIM2 Mean	PIM2 Median	PIM2 sd
Near miss	74	–4.34	–4.34	1.28
No injury (Minimal)	237	–3.79	–4.02	1.81
Intervention required (Moderate)	230	–3.72	–3.57	1.43
Patient harm or critical deterioration (Severe)	80	–3.61	–3.47	1.46

OR = odds ratio, PIM2 = Pediatric Index of Mortality 2.

All analyses were conducted using SPSS, Version 29 (IBM Corp., Armonk, NY), with *p* values of less than 0.05 considered statistically significant.

## RESULTS

### Study Population Characteristics

The baseline characteristics of the study population are summarized in **Supplemental Table 1** (https://links.lww.com/CCX/B622), providing an overview of key demographic and clinical variables that establish the foundation for temporal comparisons and risk factor analysis.

### Temporal Trends in Adverse Events

**Figure 1.**
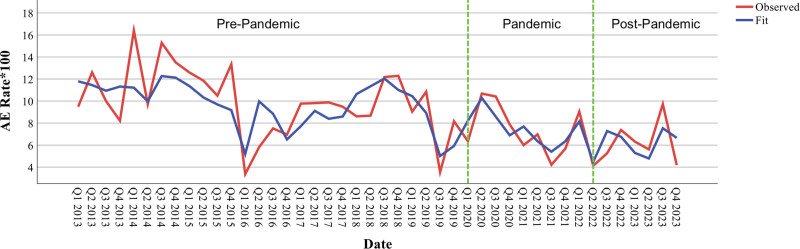
Quarterly adverse event (AE) rates in a pediatric critical care unit (PCCU) before, during, and after the COVID-19 pandemic: an interrupted time series analysis. Quarterly AE rates per 100 PCCU admissions are displayed from Q1 2013 to Q4 2023 (*n* = 7290 patients). The *red line* represents quarterly observed AE rates, and the *blue line* represents the autoregressive integrated moving average model-fitted values. *Vertical green dashed lines* demarcate the three study periods: pre-pandemic (January 2013–March 2020), pandemic (March 2020–March 2022), and post-pandemic (March 2022–December 2023).

At pandemic onset, there was a significant immediate increase of 5.20 events per 100 cases (*p* = 0.004), representing a 44% surge from baseline rates of 11.94 events per 100 cases. Following this initial spike, AE rates demonstrated a consistent quarterly decline of 0.81 events per 100 cases (*p* = 0.010), ultimately stabilizing at pre-pandemic levels by the end of the study period (**Fig. 1**). Post-pandemic, AEMS rates significantly increased by 1.94 events per 100 cases quarterly (*p* = 0.009).

### Length of Stay Patterns

**Figure 2.**
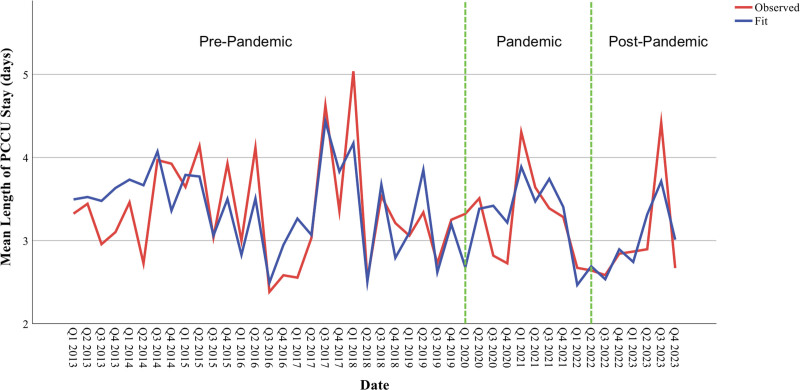
Quarterly mean length of pediatric critical care unit (PCCU) stay before, during, and after the COVID-19 pandemic: an interrupted time series analysis. Quarterly mean length of PCCU stay (days) is displayed from Q1 2013 to Q4 2023 (*n* = 7290 patients). The *red line* represents quarterly observed mean length of stay, and the *blue line* represents the autoregressive integrated moving average model-fitted values. *Vertical green dashed lines* demarcate the three study periods: pre-pandemic (January 2013–March 2020), pandemic (March 2020–March 2022), and post-pandemic (March 2022–December 2023).

Baseline LoS within the PCCU was 3.50 days. Before the pandemic, LoS demonstrated a gradual decrease of 0.01 days per quarter (*p* = 0.009), likely reflecting improving care efficiency and discharge protocols. During the pandemic period, LoS remained stable without significant trend changes (*p* = 0.684). However, post-pandemic, LoS significantly increased by 0.25 days for each additional quarter (*p* = 0.033), suggesting evolving care complexity or resource allocation patterns (**Fig. 2**).

### Predictors of Adverse Event Severity

The distribution of AE severity varied across the three COVID-19 periods, as detailed in **Supplemental Table 2** (https://links.lww.com/CCX/B622). Multinomial regression analysis revealed several unexpected findings. Neither COVID-19 period (*p* = 0.215), nor age category (*p* = 0.369) significantly predicted AE severity, indicating that pandemic-related disruptions did not fundamentally alter the nature of harm experienced by patients. Similarly, disposition category showed no significant association with AE severity (*p* = 0.811).

### The Vigilance Gradient Phenomenon

Most notably, PIM2 scores significantly predicted AE severity (*p* = 0.014), with higher PIM2 scores associated with increased odds of “MinModSev” events compared with “near miss” events. In other words, patients with lower PIM2 scores were more likely to experience “near miss” outcomes. This finding may reflect a “vigilance gradient,” whereby differences in monitoring intensity, patient physiologic reserve, or both influence the observed distribution of AE severity, rather than a true reduction in risk among higher-acuity patients. In contrast, neither PELOD scores (*p* = 0.210) nor NEMS scores (*p* = 0.368) predicted AE severity.

To further explore whether respiratory support reflected underlying patient acuity, we conducted additional exploratory analyses examining the relationship between PIM2 scores and ventilation modality. Every 1-unit increase in PIM2 was associated with a 2.85-fold increase in the odds of receiving mechanical ventilation (95% CI, 2.68–3.03; *p* < 0.001) and a more modest 1.11-fold increase in the odds of receiving noninvasive ventilation (95% CI, 1.06–1.15; *p* < 0.001; Table 1).

### Protective Effects of Respiratory Support

Mechanical ventilation (*p* = 0.039) and noninvasive ventilation (*p* = 0.015) were both significantly associated with increased odds of “MinModSev” events compared with “near miss” events, indicating a lower likelihood of “near miss” outcomes among patients receiving ventilatory support. Taken together with the PIM2-ventilation analyses, these findings suggest that patients receiving mechanical ventilation represented a distinctly higher-acuity group, whereas patients receiving noninvasive ventilation did not demonstrate comparably elevated acuity as measured by PIM2 (Table 1). Conversely, other common ICU interventions, including central venous lines (*p* = 0.502), arterial lines (*p* = 0.952), IV medications (*p* = 0.493), ICP monitors (*p* = 0.686), BiPAP (*p* > 0.995), and CPAP (*p* > 0.995), did not significantly predict AE severity.

### Clinical Severity Scores

PIM2, PELOD, and NEMS score distributions across time periods are presented in **Table 2**, demonstrating relatively stable patient acuity throughout the study period, which strengthens the validity of temporal trend comparisons.

**TABLE 2.** Pediatric Index of Mortality 2, Pediatric Logistic Organ Dysfunction, and Nine Equivalents of Nursing Manpower Use Score by Time Period

Variable	< March 2020	March 2020–March 2022	> March 2022
Pediatric Index of Mortality 2 (*n* = 4565)			
Mean	–4.44	–4.42	–4.35
sd	1.49	1.31	1.37
Pediatric Logistic Organ Dysfunction (*n* = 4588)			
Mean	5.05	4.5	4.85
sd	6.91	6.67	7.02
Nine Equivalents of Nursing Manpower Use Score (*n* = 4591)			
Mean	21.94	20.61	22.2
sd	8.17	8.06	7.7

## DISCUSSION

This study provides the first comprehensive longitudinal analysis of AE trends in a PCCU spanning nearly a decade, including the unprecedented healthcare crisis of COVID-19. Our findings reveal three critical insights with substantial implications for pediatric critical care practice and policy: COVID-19 impact (crisis adaptation and healthcare resilience), the vigilance gradient (a novel patient safety phenomenon), and respiratory support as a safety protocol.

### COVID-19 Impact: Crisis Adaptation and Healthcare Resilience

The significant increase in AE rates at pandemic onset (5.20 events/100 cases; *p* = 0.004) aligns with studies demonstrating increased strain on healthcare systems during crisis periods (9). This 44% surge from baseline likely reflects the immediate impact of resource reallocation, staffing challenges, and rapid protocol modifications inherent to pandemic response. However, the subsequent consistent quarterly decline of 0.81 events per 100 cases (*p* = 0.010) suggests progressive healthcare system adaptation over time. Rather than attributing this improvement solely to protocol or guideline development, these trends likely reflect a multifactorial process that included staffing stabilization, workflow normalization, experiential learning, and iterative refinement of care processes as teams adapted to sustained crisis conditions.

Our finding that the pandemic period was not a significant predictor of AE severity (*p* = 0.215) once the immediate crisis phase passed suggests that while AE frequency initially increased, the fundamental nature and impact of these events remained consistent. This stability may reflect the cumulative effect of system-level adaptation rather than any single intervention, including but not limited to targeted safety measures or formalized protocol (15–18). The observed trends mirror those in adult critical care settings, where resource constraints and heightened stress levels initially contributed to adverse outcomes, but systems demonstrated adaptability over time (9, 19).

Within pediatric populations, observed changes may also reflect shifts in healthcare-seeking behavior. Early in the pandemic, delayed presentations may have occurred as families avoided hospitals until illness severity necessitated urgent care (20–23). This trend could have contributed to higher AE rates due to patients presenting later in their disease course with greater acuity. Concurrently, healthcare providers adapted to evolving protocols and workflow modifications; however, AE rates increased post-pandemic, suggesting that system strain and changing care dynamics may have contributed to heightened patient safety risks during this period (24, 25).

### The Vigilance Gradient: A Novel Patient Safety Phenomenon

Our most striking and clinically significant finding is the identification of a “vigilance gradient.” Because AE severity was treated as a categorical outcome in the regression, “near miss” events were used as the reference category. The results show that higher PIM2 scores were associated with fewer near miss events relative to MinModSev events, reflecting a shift toward more severe AEs among higher-acuity patients. In contrast, lower-acuity patients, who receive less intensive surveillance, experience more near miss events. This “vigilance gradient” therefore reflects differences in monitoring and attention rather than a true reduction in risk among sicker patients.

An alternative explanation is that higher-acuity patients may have reduced physiologic reserve, such that any AE is more likely to result in measurable harm rather than a near miss. In this context, the lower frequency of near miss events among patients with higher PIM2 scores may reflect decreased patient resiliency rather than, or in addition to, increased clinical vigilance. Importantly, our data cannot distinguish whether this pattern represents a true reduction in overall AE frequency or a shift from near miss events toward the MinModSev category. As such, both increased vigilance and reduced physiologic reserve remain plausible mechanisms.

Our exploratory analyses of respiratory support modalities provide additional context for this phenomenon. The strong association between increasing PIM2 scores and mechanical ventilation confirms that mechanically ventilated patients were appropriately classified as higher-acuity, supporting the interpretation that the vigilance gradient is primarily driven by underlying illness severity. In contrast, the much weaker association between PIM2 and noninvasive ventilation suggests that NIV was frequently used in patients without substantially elevated predicted mortality risk.

One plausible explanation is that patients with lower PIM2 scores, who were more likely to experience “near miss” events, may receive relatively less intensive monitoring and attention from healthcare providers, increasing their risk for less severe but still clinically significant AEs (26). This finding has important implications for risk stratification and resource allocation in PICUs, suggesting that current monitoring protocols may need recalibration to ensure that moderate-risk patients receive sufficient surveillance to prevent progression to more severe AEs.

The absence of significant associations between PELOD scores (*p* = 0.210) or NEMS scores (*p* = 0.368) and AE severity further supports this interpretation. While PELOD reflects organ dysfunction severity and NEMS indicates nursing workload, neither appears to influence the intensive monitoring patterns that may drive the vigilance gradient effect. This distinction is crucial for developing targeted interventions and resource allocation strategies (27).

### Respiratory Support As a Safety Protocol

The association between mechanical ventilation (*p* = 0.039) and noninvasive ventilation (*p* = 0.015) with increased odds of “MinModSev” events compared with “near miss” events represents an important finding with clinical implications. When interpreted alongside PIM2-ventilation correlations, these findings suggest that the observed relationship for mechanical ventilation reflects true patient acuity, whereas the association with noninvasive ventilation likely reflects practice patterns in which lower-acuity patients receive NIV without a commensurate increase in predicted mortality risk. Rather than indicating a protective effect, this likely reflects that patients requiring respiratory support are higher-acuity and thus at greater risk for more severe AEs (28–35). These findings highlight the need for vigilant monitoring and structured care protocols for all high-risk interventions in pediatric critical care, not just those involving respiratory support.

The lack of association between other common ICU interventions and AE severity may reflect the fact that these procedures are typically performed under well-established protocols, helping to reduce associated harm (32–35). However, the specific protective effect seen with respiratory support warrants further investigation to identify transferable safety principles.

### Length of Stay Dynamics and Healthcare Adaptation

The observed LoS patterns provide additional insights into healthcare system adaptation during crisis periods. The pre-pandemic trend toward shorter stays (0.01 d quarterly decrease; *p* = 0.009) likely reflects ongoing care optimization and discharge efficiency improvements. The stability during the pandemic suggests that despite initial disruptions, core patient flow processes were maintained.

The post-pandemic increase in LoS (0.25 d quarterly increase; *p* = 0.033) may reflect several factors: increased patient complexity, more conservative discharge practices following pandemic experiences, or resource reallocation affecting discharge planning processes. This trend warrants continued monitoring to ensure it represents thoughtful care adaptation rather than inefficiency (36–42).

### Broader Context and Literature Integration

Our findings contribute to the growing understanding of AE patterns in pediatric critical care. AEs remain a persistent challenge in these settings, where care complexity inherently increases complication likelihood (3, 43–45). The pediatric population’s unique vulnerability due to developmental physiology and specialized intervention requirements makes AE prevention particularly crucial (46).

Previous research found that 62% of pediatric critical care patients experienced at least one AE, with catheter complications, uncontrolled pain, and endotracheal tube malposition being most common (1). Our findings complement this work by providing longitudinal perspective and identifying factors that influence AE severity rather than just occurrence.

The pandemic’s impact on pediatric healthcare extended beyond direct COVID-19 effects, influencing care-seeking behaviors and healthcare delivery patterns (20–25). Our study provides quantitative evidence of healthcare system resilience and adaptation, offering valuable insights for future crisis preparedness.

### Clinical Implications and Future Directions

These findings have immediate practical applications for PCCU management across multiple domains. While the observed temporal trends suggest that healthcare systems can adapt to crisis-related strain, we caution against attributing these improvements to any single strategy, such as protocol development or rapid response implementation.

Rapid response protocols may represent one potential approach to mitigating early AE surges during crises; however, their effectiveness and feasibility are highly dependent on institutional context, available resources, staffing models, and baseline safety infrastructure.

Healthcare systems should anticipate initial AE surges during crises and implement rapid response protocols to minimize duration and impact, incorporating lessons learned from COVID-19 adaptation strategies. The identification of the vigilance gradient suggests that current monitoring intensity algorithms may require recalibration to ensure adequate surveillance for moderate-risk patients who may be experiencing a “surveillance gap” due to their lower perceived acuity. Future research should validate these findings through multicenter studies and explore the mechanistic basis of the vigilance gradient through detailed process analyses, while investigating specific monitoring protocols that drive protective effects to yield actionable interventions for broader clinical application.

An important future research direction is the systematic description and evaluation of AE monitoring systems within pediatric critical care. Dedicated methodological work describing the development, implementation, and refinement of the AEMS platform—including definitions of clinically meaningful AEs, data capture processes, and lessons learned—could provide a practical framework for other institutions seeking to establish robust AE surveillance systems and identify targeted quality improvement opportunities.

### Study Limitations

Several limitations merit consideration. As a single-center retrospective analysis, results may not fully generalize to institutions with different patient populations, resources, or care models. However, our institution’s role as a regional trauma center serving a large catchment area enhances external validity. Reliance on AEMS data may underestimate true AE prevalence due to well-documented underreporting in voluntary systems (47–49). However, this limitation would be consistent across time periods, preserving the validity of trend analyses. The observational design limits causal inference, but the ITS approach strengthens our ability to attribute changes to temporal factors, particularly the pandemic period. Importantly, monitoring or surveillance intensity was not directly measured in this study. Rather, differences in monitoring were inferred based on established ICU practice patterns, patient acuity scores (including PIM2), and the burden of critical care interventions. Future research should validate these findings in multicenter studies and explore the mechanistic basis of the vigilance gradient through detailed process analyses. Investigation of specific monitoring protocols that drive protective effects could yield actionable interventions for broader application.

## CONCLUSIONS

This study demonstrates that pediatric critical care systems showed increased AE rates post-pandemic, reflecting the impact of system strain and evolving care dynamics during this period. The identification of a novel “vigilance gradient,” in which higher-acuity patients had lower odds of near miss events highlights the impact of patient acuity on monitoring intensity and has important implications for risk-stratified safety strategies. The association of respiratory support with higher odds of more severe events suggests that patients requiring these interventions are higher-acuity, highlighting the need for systematic care approaches to ensure adequate monitoring and potentially improve safety outcomes across all high-risk patient groups. These findings establish evidence-based frameworks for crisis preparedness, risk-stratified monitoring, and targeted safety interventions in pediatric critical care. Most importantly, this research provides actionable intelligence for PCCU administrators and clinicians: crisis preparedness protocols should anticipate and plan for initial AE surges, monitoring intensity algorithms may need recalibration to address potential surveillance gaps for moderate-risk patients, and successful safety protocols from respiratory care warrant systematic analysis and broader application. As pediatric critical care continues to evolve, these insights provide a foundation for evidence-based improvements in patient safety, crisis preparedness, and outcome optimization for our most vulnerable patients.

## Supplementary Material
