# The Vigilance Gradient: Eleven Years of Adverse Event Trends in Pediatric Critical Care: Erratum

**DOI:** 10.1097/CCE.0000000000001446

**Published:** 2026-07-22

**Authors:** Devika Singh, Michael R. Miller, Maitray A. Patel, Cory Anderson, Douglas D Fraser

In the article appearing in volume 8, issue 5 of Critical Care Explorations, the title of the article contained an error.

The title appeared incorrectly as: “The Vigilance Gradient: Nine Years of Adverse Event Trends in Pediatric Critical Care”.

The title has been corrected in the online and PDF versions to “The Vigilance Gradient: Eleven Years of Adverse Event Trends in Pediatric Critical Care”.
