# Supplementary material for: The Vigilance Gradient: Eleven Years of Adverse Event Trends in Pediatric Critical Care
Source: Crit Care Explor. 2026 Apr 24;8(5):e1407. doi: 10.1097/CCE.0000000000001407 (PMC13120637; doi:10.1097/CCE.0000000000001407)
Supplement: Supplementary file 1 [file cc9-8-e1407-s001.pdf]

SUPPLEMENTAL TABLES

TABLE OF CONTENTS

Page 3-4: Supplemental Table 1: Patient Demographics and Other Select Variables by Adverse Event Occurrence.

Page 5-6: Supplemental Table 2: Frequencies of Select Variables by Time Period.

Supplemental Table 1: Patient Demographics and Other Select Variables by Adverse Event Occurrence

Characteristics	PCCU AE		No PCCU AE	
	n	%	*n*	%
**Gender**				
Female	280	44.9%	2924	43.9%
Male	344	55.1%	3741	56.1%
**Age**				
Infancy	284	45.5%	2520	37.8%
Toddler/Early Childhood	122	19.6%	1372	20.6%
Middle Childhood	74	11.9%	1205	18.1%
Adolescence	144	23.1%	1569	23.5%
**Disposition Category**				
Died	46	7.4%	160	2.4%
Discharge	471	75.6%	5692	85.4%
Transfer Inpatient	54	8.7%	446	6.7%
Transfer Another	14	2.2%	63	0.9%
Transfer Acute	37	5.9%	293	4.4%
LAMA	1	0.2%	10	0.2%
**Central Venous Line**				
No	275	44.1%	4788	71.8%
Yes	349	55.9%	1878	28.2%
**Arterial Line**				
No	361	57.9%	5198	78.0%
Yes	263	42.1%	1468	22.0%
**IV Medications**				
No	26	4.2%	1087	16.3%
Yes	598	95.8%	5579	83.7%
**ICP Monitoring**				
No	586	93.9%	6554	98.3%
Yes	38	6.1%	112	1.7%
**Mechanical Ventilation**				

No	257	41.2%	4591	68.9%
Yes	367	58.8%	2075	31.1%
**Non-Invasive Ventilation**				
No	384	61.5%	4657	69.9%
Yes	240	38.5%	2009	30.1%
**BiPAP**				
No	662	99.7%	6641	99.6%
Yes	2	0.3%	25	0.4%
**CPAP**				
No	662	99.7%	6640	99.6%
Yes	2	0.3%	26	0.4%
**HFNC**				
No	617	98.9%	6586	98.8%
Yes	7	1.1%	80	1.2%
**Multiple IV Medications**				
No	549	88.0%	6413	96.2%
Yes	75	12.0%	253	3.8%

Supplemental Table 2: Frequencies of Select Variables by Time Period

Variable	< Mar 2020		Mar 2020-Mar 2022		> Mar 2022	
	n	*%*	*n*	*%*	*n*	*%*
**Adverse Event Severity**						
Near Miss	55	12.4%	10	10.4%	9	10.8%
No Injury (Minimal Event)	177	39.8%	31	32.3%	31	37.3%
Intervention required (Moderate Event)	160	36.0%	44	45.8%	26	31.3%
Patient harm or clinical deterioration (Severe Event)	53	11.9%	11	11.5%	17	20.5%
**Disposition Category**						
Died	135	2.9%	34	2.6%	37	2.7%
Discharge	3907	85.1%	1096	83.7%	1160	83.5%
Transfer Inpatient	149	3.2%	171	13.1%	180	13.0%
Transfer Another	66	1.4%	6	0.5%	5	0.4%
Transfer Acute	330	7.2%	0	0.0%	0	0.0%
LAMA	4	0.1%	3	0.2%	4	0.3%
**Age Category**						
Infancy	1870	40.7%	460	35.1%	474	34.1%
Toddler/Early Childhood	911	19.8%	235	17.9%	348	25.1%
Middle Childhood	793	17.3%	242	18.5%	244	17.6%
Adolescence	1017	22.2%	373	28.5%	323	23.3%
**Central Venous Line**						
No	3088	67.3%	923	70.5%	1052	75.7%
Yes	1503	32.7%	387	29.5%	337	24.3%
**Arterial Line**						
No	3470	75.6%	1033	78.9%	1056	76.0%
Yes	1121	24.4%	277	21.1%	333	24.0%
**Other IV Medications**						
No	669	14.6%	255	19.5%	189	13.6%
Yes	3922	85.4%	1055	80.5%	1200	86.4%

**ICP Monitoring**						
No	4495	97.9%	1293	98.7%	1352	97.3%
Yes	96	2.1%	17	1.3%	37	2.7%
**Mechanical Ventilation**						
No	2956	64.4%	948	72.4%	944	68.0%
Yes	1635	35.6%	362	27.6%	445	32.0%
**Non-Invasive Ventilation**						
No	3261	71.0%	944	72.1%	836	60.2%
Yes	1330	29.0%	366	27.9%	553	39.8%
**BiPAP**						
No	4591	100.0%	1310	100.0%	1362	98.1%
Yes	0	0.0%	0	0.0%	27	1.9%
**CPAP**						
No	4591	100.0%	1310	100.0%	1361	98.0%
Yes	0	0.0%	0	0.0%	28	2.0%
**HFNC**						
No	4590	100.0%	1310	100.0%	1303	93.8%
Yes	1	0.0%	0	0.0%	86	6.2%
**Multiple IV Medications**						
No	4374	95.3%	1259	96.1%	1329	95.7%
Yes	217	4.7%	51	3.9%	60	4.3%
